# Metabolomic profiling refines cardiovascular risk stratification beyond SCORE2-diabetes in patients with concurrent type 2 diabetes and pulmonary dysfunction

**DOI:** 10.3389/fendo.2026.1933396

**Published:** 2026-08-26

**Authors:** Meili Li, Yanyan Shen, Youwei Huang, Lijun Liu, Wenkai Bin

**Affiliations:** 1 Cardiac Function Department, The Affiliated Nanhua Hospital, Hengyang Medical School, University of South China, Hengyang, China; 2 Department of Ultrasound Medicine, The Affiliated Nanhua Hospital, Hengyang Medical School, University of South China, Hengyang, China; 3 Department of Infections, The Affiliated Nanhua Hospital, Hengyang Medical School, University of South China, Hengyang, China; 4 Department of Cardiovascular Medicine, Foshan Fosun Chancheng Hospital, Foshan, Guangdong, China; 5 Animal Injury and Poisoning Treatment Center, The Affiliated Nanhua Hospital, Hengyang Medical School, University of South China, Hengyang, China

**Keywords:** cardiovascular risk stratification, ensemble machine learning, metabolomic signature, NMR metabolomics, pulmonary dysfunction, SCORE2-diabetes, type 2 diabetes

## Abstract

**Background:**

Patients presenting with both type 2 diabetes (T2D) and lung function impairment (LFI) are exposed to an amplified risk of major adverse cardiovascular events (MACE). Although the SCORE2-Diabetes algorithm is widely endorsed for clinical risk assessment, its prognostic accuracy within this specific multimorbid phenotype remains inadequately explored. This study sought to evaluate the baseline utility of SCORE2-Diabetes and to investigate whether integrating a novel, machine learning-derived metabolomic signature could optimize 10-year MACE prediction in this highly vulnerable population.

**Methods:**

We conducted a prospective analysis of UK Biobank participants with type 2 diabetes (T2D) and no cardiovascular disease at baseline. A reference cohort of 12,130 participants with preserved lung function was used to assess the base clinical model, and the primary cohort comprised 3,706 participants with lung function impairment (LFI). We profiled 249 plasma metabolites using nuclear magnetic resonance (NMR) spectroscopy. A machine-learning framework combining least absolute shrinkage and selection operator (LASSO) Cox regression, random forest survival analysis, and extreme gradient boosting (XGBoost) was used to derive a consensus signature. Internal validation used 1,000 bootstrap resamples; the complete modeling pipeline was repeated within each resample. Incremental performance beyond SCORE2-Diabetes was assessed using Harrell’s concordance index (C-index), net reclassification improvement (NRI), calibration, and decision curve analysis (DCA).

**Results:**

During a median follow-up of 12 years, SCORE2-Diabetes showed lower discrimination in the LFI cohort than in the reference cohort (C-index, 0.670 vs. 0.707). Among the 3,706 participants with LFI, 845 developed major adverse cardiovascular events (MACE). Adding the 12-metabolite signature increased the C-index from 0.670 to 0.722 (absolute difference, 0.052; P < 0.001), representing a statistically significant but moderate improvement in discrimination. The five-metabolite model had a C-index of 0.708, while the two-metabolite sensitivity model yielded a C-index of 0.699 (95% CI, 0.683-0.716). Using the 2023 European Society of Cardiology SCORE2-Diabetes categories, the categorical NRI was 14.8% (95% CI, 10.8%-18.8%). Internal calibration and DCA suggested improved agreement and net benefit, but these results require external validation.

**Conclusions:**

In participants with T2D and LFI, adding a metabolomic signature to SCORE2-Diabetes produced a statistically significant but moderate improvement in risk discrimination. These internally validated findings support further evaluation in independent cohorts and prospective impact studies; they do not establish immediate clinical usefulness.

## Introduction

Type 2 diabetes (T2D) represents a global health crisis, primarily due to its strong association with cardiovascular disease (CVD), which remains the leading cause of morbidity and mortality in this population (1, 2). Accurate risk stratification is paramount for guiding preventive strategies, yet conventional risk prediction models often fall short (3). The recently developed SCORE2-Diabetes algorithm, while a significant advancement, may not fully capture the heterogeneous risk profiles observed in clinical practice, particularly in patients with complex comorbidities (4, 5). This highlights an unmet need for more refined risk assessment tools that incorporate novel biomarkers to improve upon existing clinical scores.

Emerging evidence has established a compelling link between impaired lung function and an elevated risk of cardiovascular events, especially in individuals with T2D (6). Both restrictive and obstructive lung patterns, which are highly prevalent in the T2D population, are independently associated with increased cardiovascular mortality (7). This connection suggests a shared pathophysiological axis, possibly mediated by systemic inflammation, oxidative stress, and insulin resistance, which links pulmonary dysfunction to adverse cardiovascular outcomes (8–10). However, the incremental predictive value of lung function impairment is not accounted for in current risk algorithms, leaving a critical gap in the risk stratification of this high-risk subgroup.

Metabolomics, the large-scale study of small molecules, offers a powerful lens to capture the downstream effects of genetic and environmental factors on an individual’s phenotype, providing a real-time snapshot of their physiological state (11). Circulating metabolites have shown immense promise for enhancing CVD risk prediction beyond traditional risk factors (12). Specific metabolic signatures, such as those involving branched-chain amino acids, inflammatory glycoproteins, and lipid species, have been identified as key players in the pathogenesis of both T2D and CVD (13, 14). Leveraging these metabolic insights could therefore provide a more granular and dynamic assessment of cardiovascular risk.

**Figure 1:**
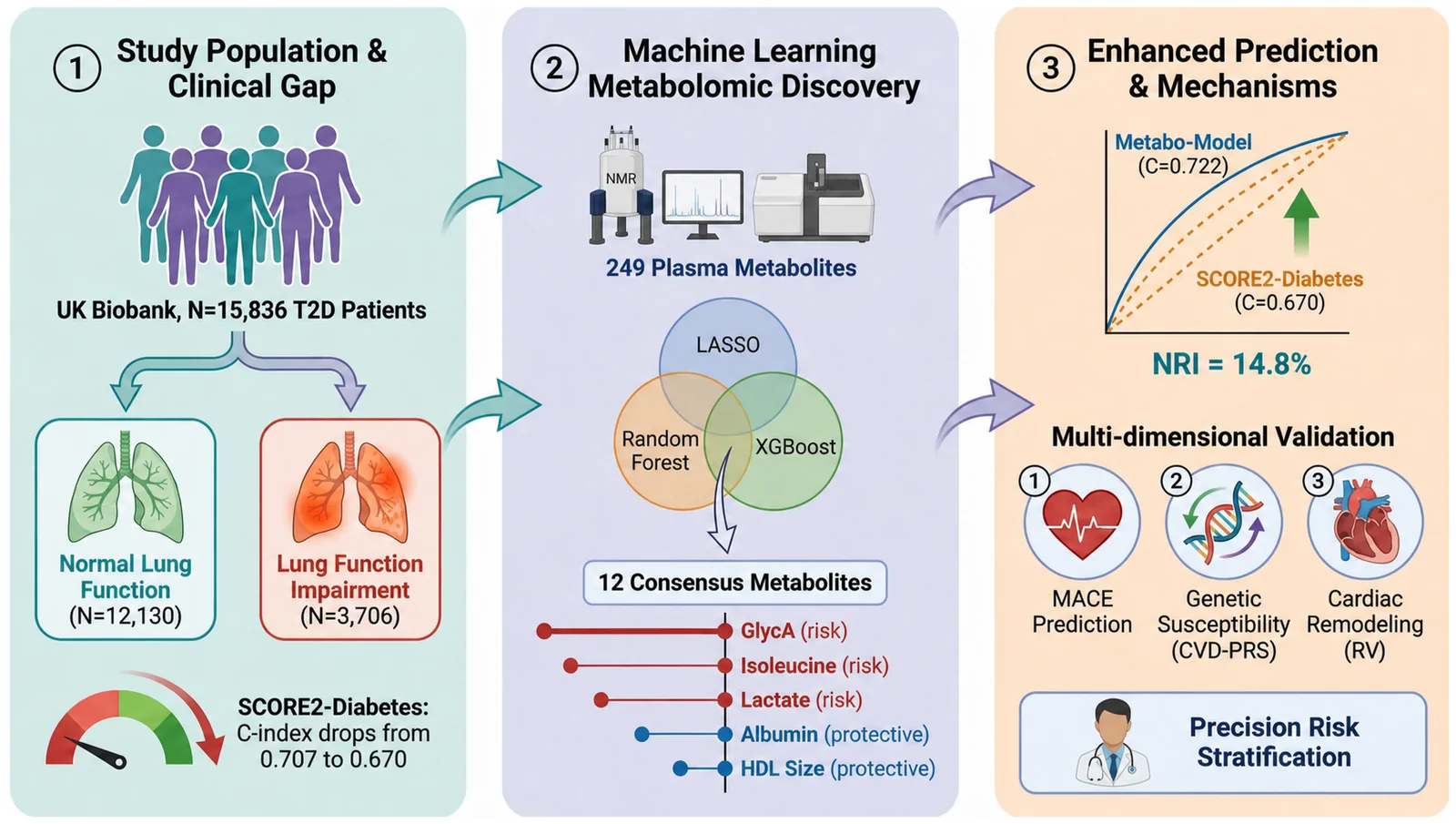
Graphical abstract. Overview of the study population, ensemble machine-learning metabolomic discovery, improvement in internal predictive performance, and multidimensional associations with MACE, genetic susceptibility, and cardiac remodeling. C-index, concordance index; CVD-PRS, cardiovascular disease polygenic risk score; LFI, lung function impairment; MACE, major adverse cardiovascular events; NMR, nuclear magnetic resonance; NRI, net reclassification improvement; RV, right ventricle; T2D, type 2 diabetes.

In this study, we hypothesized that a machine learning-derived metabolic signature could significantly improve the prediction of 10-year MACE risk in a high-risk cohort of individuals with T2D and concomitant lung function impairment, beyond the established SCORE2-Diabetes model. We aimed to: 1) identify a robust metabolic signature for MACE using multiple machine learning algorithms; 2) validate its multi-dimensional associations with clinical outcomes, genetic predisposition, and cardiac structure; and 3) quantify its incremental value in risk discrimination, reclassification, and clinical utility. A graphical overview of the study population, metabolomic discovery framework, predictive evaluation, and multidimensional validation is presented in **Figure 1**.

## Methods

### Study participants

This prospective cohort study was conducted using data from the UK Biobank (UKB), a large-scale, population-based resource with over 500,000 participants aged 40–69 years recruited between 2006 and 2010 across the United Kingdom (15). The UKB has ethical approval from the North West Multi-center Research Ethics Committee (11/NW/0382). All participants provided written informed consent.

The selection of the study cohort is illustrated in **Supplementary Figure 1**. From the total UKB cohort, we identified participants with T2D who were free of major adverse cardiovascular events (MACE) at baseline. We then focused on a high-risk subgroup characterized by LFI. After applying exclusion criteria, the final analytical cohort comprised 3,706 individuals with T2D and LFI, with a reference group of 12,130 individuals with T2D and preserved lung function.

### Ascertainment of type 2 diabetes

T2D status at baseline was ascertained using a composite algorithmic approach designed to maximize diagnostic accuracy (16). This method integrated self-reported medical history, specific use of glucose-lowering medications (including insulin), and hospital inpatient records coded according to the International Classification of Diseases (ICD-10). The specific algorithms, field IDs, and diagnostic codes utilized are listed in **Supplementary Table 1**.

### Assessment of lung function

Lung function was assessed at baseline using standard spirometry. To account for demographic variations, we utilized the Global Lung Function Initiative (GLI) 2012 reference equations to derive the lower limit of normal (LLN) (6, 17). Participants were categorized into “Preserved Lung Function” or “Lung Function Impairment” based on their forced expiratory volume in 1 second (FEV1) and forced vital capacity (FVC). LFI was further sub-phenotyped into obstructive and restrictive physiology. Detailed protocols for spirometry quality control and precise diagnostic criteria for each physiological category are described in **Supplementary Methods** and **Supplementary Table 2**.

### Ascertainment of clinical outcomes

The primary endpoint was the incidence of MACE, defined as a composite of non-fatal myocardial infarction, non-fatal stroke, and cardiovascular death (18). Outcomes were tracked through linkage to national hospital admission databases and death registries. Participants were followed from recruitment until the first MACE event, death from other causes, or the end of the follow-up period. Complete definitions and ICD-10 codes for these endpoints are provided in **Supplementary Table 3**.

In the primary cause-specific Cox analyses, deaths from non-cardiovascular causes were censored at the date of death. Sensitivity analyses treated non-cardiovascular death as a competing event using Fine-Gray subdistribution hazards models and the Aalen-Johansen estimator (**Supplementary Table 11**).

### SCORE2-diabetes model

To establish a clinical baseline for risk comparison, we calculated the SCORE2-Diabetes risk score for all eligible participants. This algorithm incorporates conventional risk factors including age, smoking status, systolic blood pressure, total cholesterol, high-density lipoprotein cholesterol (HDL-C), age at diabetes diagnosis, glycated hemoglobin (HbA_1c_), and estimated glomerular filtration rate (eGFR) (4). Detailed calculation methods and variable definitions are available in the **Supplementary Methods**.

Current smoking was defined from UK Biobank field 20116 as self-reported current smoking at baseline; former and never smokers were classified as non-current smokers.

### Profiling of plasma metabolomics

Circulating metabolic biomarkers were quantified in baseline plasma samples using a high-throughput proton NMR spectroscopy platform (Nightingale Health Ltd.) (19). This panel provides simultaneous quantification of 249 metabolic measures, covering routine lipids, lipoprotein subclasses, fatty acids, and low-molecular-weight metabolites. Details regarding sample handling, quality control, and the full list of measured metabolites are provided in the **Supplementary Methods**.

### Polygenic risk score

To explore genetic susceptibility, we utilized the Standardized Polygenic Risk Score for CVD (CVD-PRS) provided by the UK Biobank. This score was generated using a Bayesian approach integrating summary statistics from large-scale genome-wide association studies, accounting for approximately 1.1 million genetic variants (20).

The cardiovascular disease polygenic risk score (CVD-PRS) analysis included 3,488 participants, of whom 797 experienced MACE. Baseline characteristics of participants with and without available CVD-PRS data were compared using standardized mean differences (**Supplementary Table 7**).

### Assessment of cardiac phenotypes

Cardiac structural and functional parameters were derived from cardiovascular magnetic resonance (CMR) imaging in a subset of participants (21). Key phenotypes, including left ventricular mass (LVM), left ventricular end-diastolic volume (LVEDV), and right ventricular end-diastolic volume (RVEDV), were analyzed to investigate potential pathophysiological mechanisms linking metabolic alterations to heart remodeling.

The cardiovascular magnetic resonance (CMR) analysis included 812 participants, of whom 111 experienced MACE. We compared the CMR subset with the full LFI cohort using standardized mean differences to assess potential selection into imaging (**Supplementary Table 7**).

### Statistical analyses

**Figure 2:**
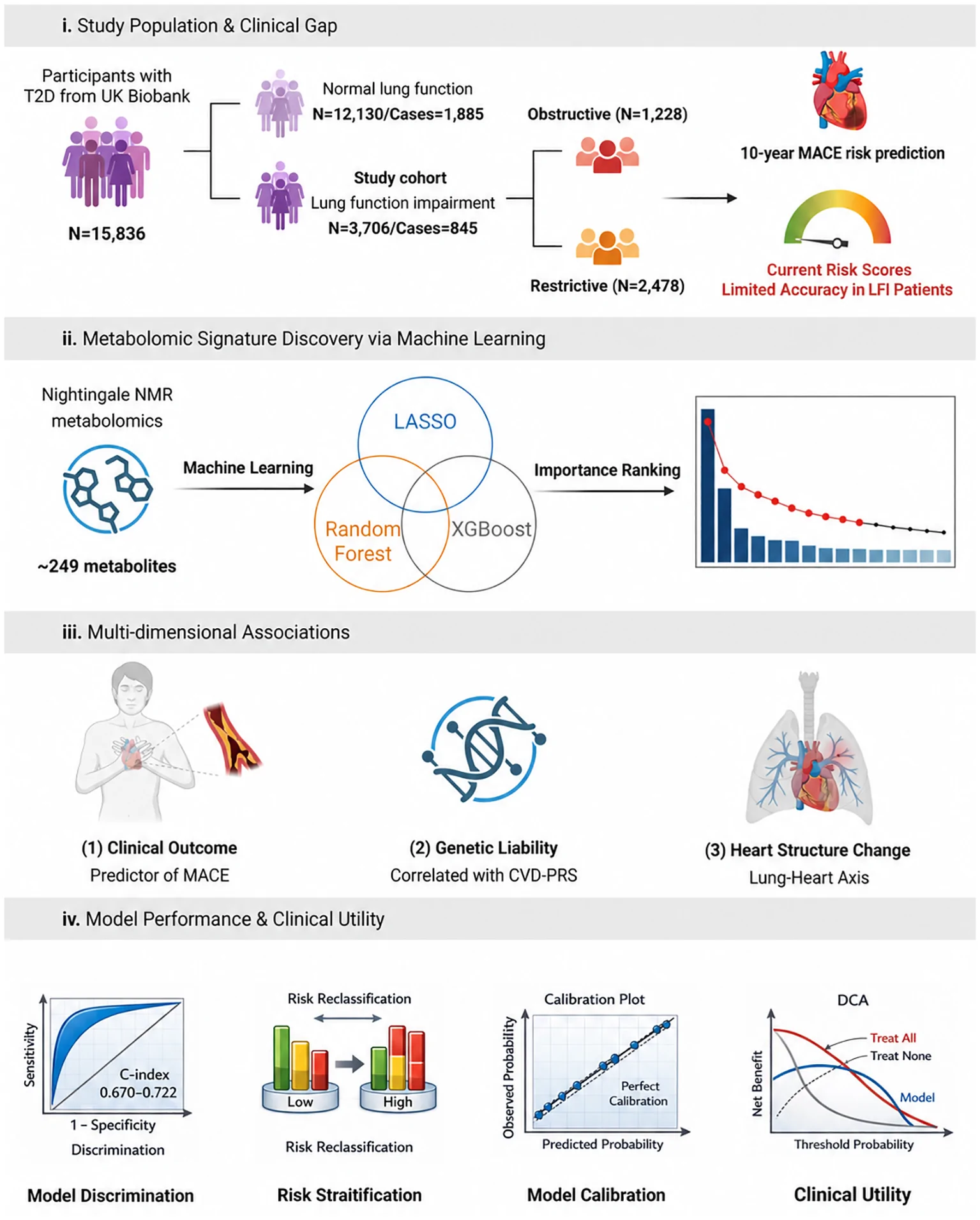
Study design and analytical workflow. Overview of cohort selection, NMR metabolomic profiling, consensus feature selection, multidimensional association analyses, and evaluation of discrimination, reclassification, calibration, and decision-curve performance. The discrimination panel reports the observed C-index range of 0.670-0.722. CVD, cardiovascular disease; DCA, decision curve analysis; LFI, lung function impairment; MACE, major adverse cardiovascular events; NMR, nuclear magnetic resonance; PRS, polygenic risk score; T2D, type 2 diabetes.

The comprehensive analytical workflow is depicted in **Figure 2**. All statistical analyses were conducted using R software (version 4.5.0). A two-sided P-value < 0.05 was considered statistically significant. Missing values for clinical covariates were minimal and imputed using a random forest-based algorithm (**Supplementary Table 4**) (22).

For internal validation, we used 1,000 bootstrap resamples. Within each resample, missing-data imputation, metabolite standardization, hyperparameter tuning, feature selection, and model fitting were repeated *de novo*. Tuning was performed by inner 10-fold cross-validation, and performance was evaluated in out-of-bag observations before aggregation across resamples. The random forest tuning grid was mtry {8, 16, 24, 32, 48}, minimum node size {5, 10, 20}, and 1,000 trees; the XGBoost tuning grid was maximum depth {2, 3, 4}, learning rate {0.01, 0.05, 0.10}, minimum child weight {1, 5, 10}, subsample {0.7, 0.9, 1.0}, column subsample {0.6, 0.8, 1.0}, and up to 1,000 rounds with early stopping. The prespecified random seed was 20260802. Selection frequencies and the complete tuning specifications are reported in **Supplementary Tables 8** and **S13**, respectively.

### Machine learning-based feature selection

To identify robust metabolic predictors specifically associated with MACE in the LFI population, we implemented an ensemble feature-selection strategy to mitigate overfitting and multicollinearity. We triangulated results from three algorithms: (1) least absolute shrinkage and selection operator (LASSO) Cox regression with 10-fold cross-validation (23); (2) random survival forests, ranked by permutation variable importance (24); and (3) extreme gradient boosting (XGBoost), assessed using Gain scores (25). Only metabolites selected by all three algorithms were retained as the consensus metabolic signature for downstream modeling (**Supplementary Table 5**).

We retained the intersection of the three algorithms because they have different inductive biases and measures of importance; agreement across all three was used to prioritize reproducibility and reduce algorithm-specific false-positive selection. This conservative rule can reduce sensitivity, and we therefore report method-specific selections and bootstrap selection frequencies in the **Supplementary Material**.

The top-five panel was defined at the empirical elbow of the ranked importance curve, corresponding to an importance score of approximately 0.020. Because GlycA and isoleucine had substantially greater importance than the remaining metabolites, we also evaluated a two-metabolite sensitivity model containing only these markers.

### Model development and validation

We evaluated three metabolomic-augmented models: a Full Metabo-Model incorporating all 12 consensus metabolites, a Parsimonious Metabo-Model restricted to the top five metabolites at the empirical importance-curve elbow, and a two-metabolite sensitivity model containing GlycA and isoleucine. Model coefficients are provided in **Supplementary Table 6**. Predictive performance was benchmarked against SCORE2-Diabetes (4). Discrimination was quantified using Harrell’s C-index. The 2023 European Society of Cardiology SCORE2-Diabetes categories of <5%, 5% to <10%, 10% to <20%, and ≥20% were applied (26). Incremental predictive value was assessed using categorical net reclassification improvement (NRI) and integrated discrimination improvement (IDI) (27). Model calibration was evaluated at 10 years, and decision curve analysis (DCA) estimated net benefit across threshold probabilities of 1%-30% (28). Nested-resampling performance and reclassification results are reported in **Supplementary Tables 9** and **S10**.

Calibration was assessed at 10 years by grouping participants into deciles of predicted risk. Mean predicted risk was plotted against observed cumulative incidence estimated with the Aalen-Johansen estimator, with non-cardiovascular death treated as a competing event and 95% confidence intervals obtained by bootstrap resampling. Bootstrap-corrected calibration-in-the-large, calibration slope, and Brier score were calculated for each model. DCA was evaluated over threshold probabilities of 1%-30%; net benefit was interpreted as exploratory because it depends on the selected threshold and the relative consequences assigned to false-positive and false-negative classifications.

### Association and pathway analysis

Multivariable Cox proportional hazards models were used to estimate hazard ratios (HRs) for associations between the identified metabolites and incident MACE. Multivariable linear regression was used to examine associations of metabolic signatures with genetic liability (CVD-PRS) and CMR-derived cardiac structural phenotypes (21).

The proportional hazards assumption was formally assessed using scaled Schoenfeld residuals for each covariate and a global test (**Supplementary Table 12**).

Baseline chronic liver disease was defined from linked hospital records as an ICD-10 K70-K77 diagnosis recorded before baseline, excluding acute hepatic failure (K72.0). Heterogeneity was assessed using a product term between the standardized 12-metabolite score and chronic liver disease status in the Cox model.

## Results

### Baseline characteristics of the study population

The baseline characteristics of the study cohort are presented in **Table 1**. Among 3,706 participants with T2D and lung function impairment, 845 (22.8%) developed incident MACE over a median follow-up of 12 years. Participants in the MACE group were older (68.9 vs. 65.5 years), more likely to be male (73.7% vs. 62.4%), and the prevalence of current smoking was 12.5% (105/842) versus 9.5% (270/2,853) in those with and without MACE, respectively (P = 0.011). In addition, the MACE group was diagnosed with diabetes at a younger age (51.8 vs. 55.4 years), indicating a longer duration of disease exposure. Clinically, those who developed MACE exhibited a worse cardiometabolic profile, characterized by higher systolic blood pressure and HbA1c levels, alongside lower HDL-C concentrations and eGFR. Regarding lung function, spirometric parameters (FEV1 and FVC % predicted) were significantly lower in the MACE group, although restrictive physiology remained the dominant phenotype in both groups.

**Table 1:** Baseline clinical characteristics of type 2 diabetes patients with lung function impairment.

Variable	Total (n=3706)	No MACE (n=2861)	Incident MACE (n=845)	P-value
SCORE2-diabetes components				
Age, years	66.3 (7.9)	65.5 (8.0)	68.9 (7.1)	<0.001
Sex				<0.001
Male	2,409 (65.0%)	1,786 (62.4%)	623 (73.7%)	
Female	1,297 (35.0%)	1,075 (37.6%)	222 (26.3%)	
Systolic BP, mmHg	136.6 (16.7)	134.5 (15.6)	143.8 (18.2)	<0.001
HbA_1c_, mmol/mol	54.6 (10.6)	53.1 (9.8)	59.4 (11.7)	<0.001
eGFR, mL/min/1.73m^2^	80.3 (20.2)	84.1 (17.9)	67.5 (22.3)	<0.001
Total Cholesterol, mmol/L	4.9 (1.0)	4.8 (1.0)	5.2 (1.1)	<0.001
HDL-C, mmol/L	1.3 (0.4)	1.3 (0.4)	1.1 (0.3)	<0.001
Current Smoker, n (%)	375/3,695 (10.1%)	270/2,853 (9.5%)	105/842 (12.5%)	0.011
Age at diabetes diagnosis, years	54.6 (9.2)	55.4 (9.1)	51.8 (9.0)	<0.001
Lung function				
Physiology Type				0.228
Obstructive	1,228 (33.1%)	963 (33.7%)	265 (31.4%)	
Restrictive	2,478 (66.9%)	1,898 (66.3%)	580 (68.6%)	
FEV1, % predicted	75.9 (15.1)	78.3 (14.0)	67.9 (15.8)	<0.001
FVC, % predicted	78.2 (13.5)	80.0 (12.7)	72.0 (14.2)	<0.001

Continuous variables are presented as mean ± SD, categorical variables are reported as counts (percentages).

BP, blood pressure; eGFR, estimated glomerular filtration rate; FEV1, forced expiratory volume in 1 second; FVC, forced vital capacity; HbA_1c_, glycated hemoglobin; HDL-C, high-density lipoprotein cholesterol; MACE, major adverse cardiovascular events.

Current smoking was defined from UK Biobank field 20116 as self-reported current smoking at baseline. There were 11 participants with missing smoking status (three with MACE and eight without MACE).

### Identification and prioritization of metabolic predictors

**Figure 3:**
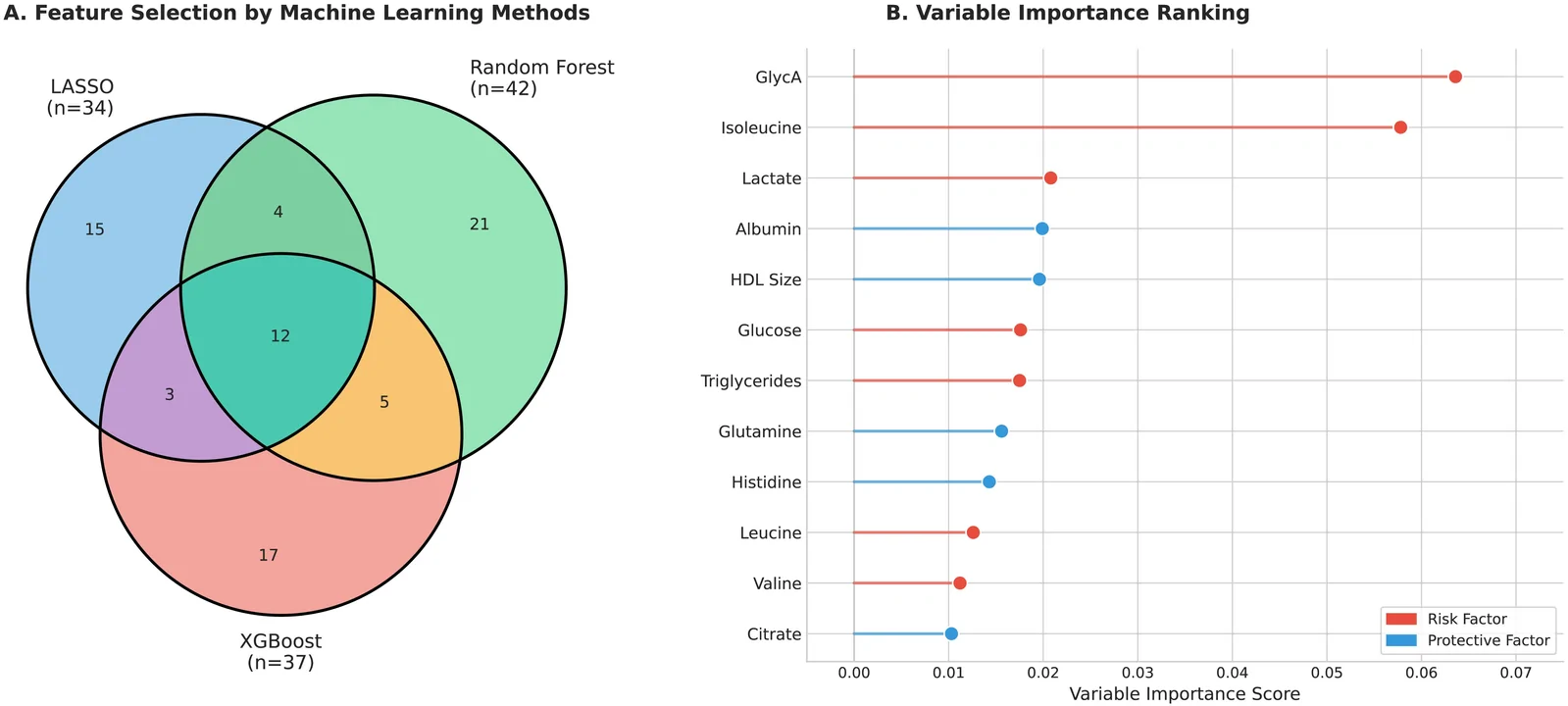
Selection and prioritization of metabolic predictors for incident MACE using ensemble machine learning approaches. **(A)** Venn diagram illustrating the multi-algorithm feature screening strategy. To identify robust metabolic signatures, three independent machine learning algorithms were applied: LASSO regression, RF, and XGBoost. **(B)** Variable importance ranking of the selected metabolic signatures. The chart displays the relative predictive contribution of each metabolite. The five-metabolite panel corresponds to the empirical elbow at an importance score of approximately 0.020; the two dominant predictors (GlycA and isoleucine) were additionally evaluated as a sensitivity model. GlycA, glycoprotein acetyls; LASSO, Least Absolute Shrinkage and Selection Operator; MACE, major adverse cardiovascular events; RF, Random Forest; XGBoost, Extreme Gradient Boosting.

To identify robust metabolic signatures associated with MACE in the context of LFI, we employed an ensemble machine learning strategy integrating LASSO, Random Forest, and XGBoost algorithms (**Figure 3A**). This approach yielded a consensus panel of 12 metabolites consistently selected by all three methods. The identified signatures encompassed inflammatory and fluid balance markers (GlycA, Albumin), branched-chain amino acids (Isoleucine, Leucine, Valine), energy metabolism intermediates (Lactate, Glucose, Citrate), lipid parameters (Triglycerides, HDL particle size), and other amino acids (Glutamine, Histidine). Variable importance analysis ranked GlycA and Isoleucine as the top contributors to predictive accuracy, followed by Lactate, Albumin and HDL size (**Figure 3B**).

GlycA and isoleucine had markedly greater importance than the remaining metabolites, motivating the prespecified two-metabolite sensitivity model described below.

### Multi-dimensional associations with outcomes, genetics, and cardiac structure

**Figure 4:**
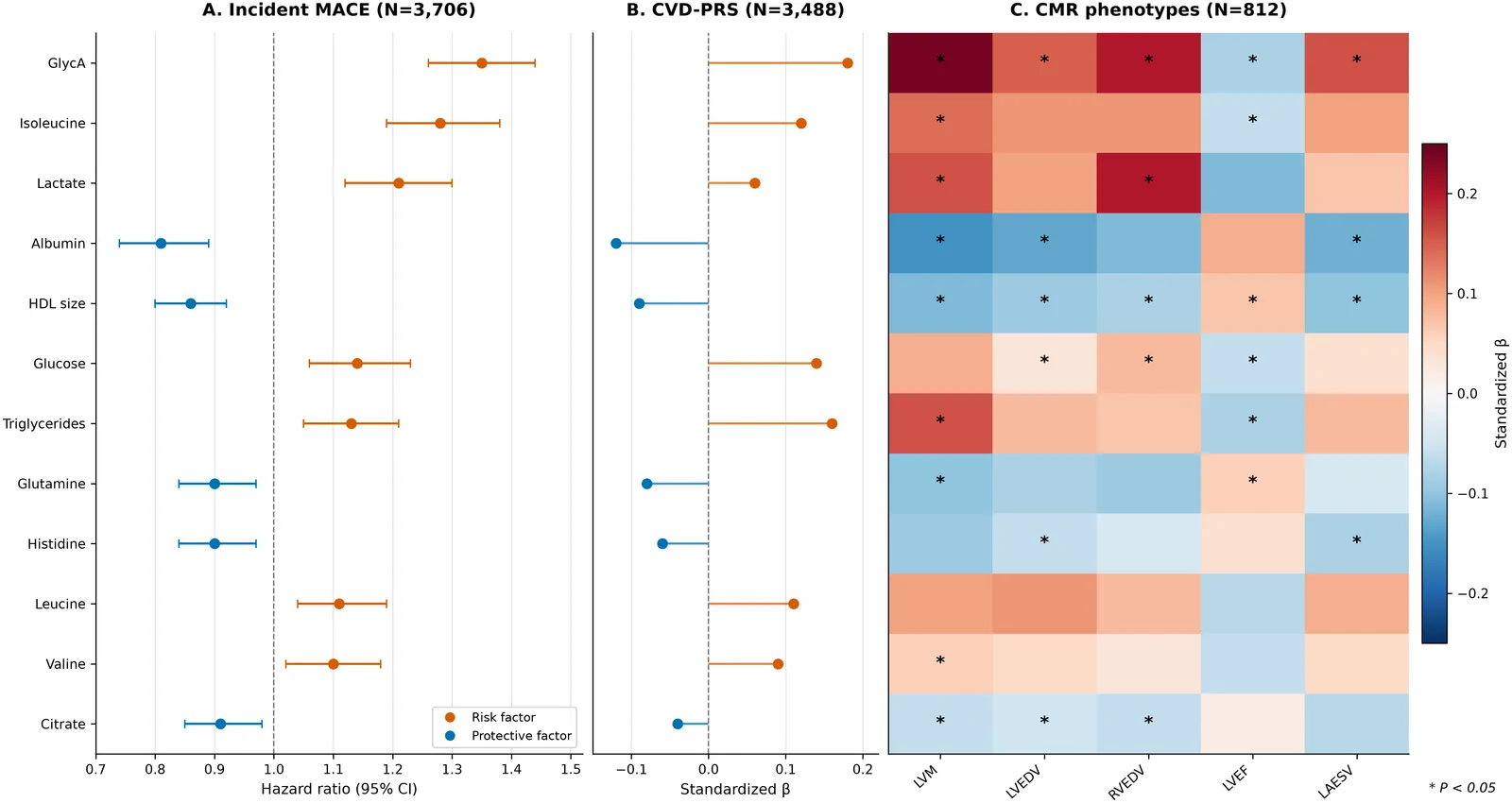
Multidimensional associations of identified metabolic signatures with clinical outcomes, genetic susceptibility, and cardiac remodeling. **(A)** Associations of the 12 consensus metabolites with incident MACE in the LFI cohort (N = 3,706). **(B)** Associations with CVD-PRS in 3,488 participants. **(C)** Associations with CMR phenotypes in 812 participants. HDL particle size is included among the 12 consensus metabolites. Color intensity represents standardized beta coefficients; red indicates positive and blue indicates inverse associations. Asterisks denote P<0.05. CI, confidence interval; CVD-PRS, cardiovascular disease polygenic risk score; GlycA, glycoprotein acetyls; HR, hazard ratio; LAESV, left atrial end-systolic volume; LVEDV, left ventricular end-diastolic volume; LVEF, left ventricular ejection fraction; LVM, left ventricular mass; MACE, major adverse cardiovascular events; RVEDV, right ventricular end-diastolic volume.

Multivariable Cox proportional hazards regression confirmed independent associations between the selected metabolic signatures and incident MACE (**Figure 4A**). Among the identified predictors, the inflammatory marker GlycA demonstrated the strongest association with adverse outcomes (HR per 1-SD increase: 1.35; 95% CI: 1.26–1.44), followed by the branched-chain amino acid Isoleucine (HR: 1.28; 95% CI: 1.19–1.38) and the glycolysis product Lactate (HR: 1.21; 95% CI: 1.12–1.30). Conversely, higher levels of Albumin (HR: 0.81; 95% CI: 0.74–0.89) and larger HDL particle size (HR: 0.86; 95% CI: 0.80–0.92) were significantly associated with a reduced risk of MACE.

To elucidate the biological mechanisms driving these associations, we examined correlations with genetic susceptibility and cardiac structure. We observed significant positive correlations between the CVD-PRS and key risk metabolites, particularly GlycA (r = 0.18, P < 0.001) and Isoleucine (**Figure 4B**). Furthermore, analyses of CMR phenotypes showed that these metabolic perturbations were associated with adverse cardiac remodeling (**Figure 4C**). Specifically, GlycA was positively associated with left ventricular mass (standardized β=0.24), and lactate was positively associated with right ventricular end-diastolic volume (standardized β=0.20); albumin showed inverse associations with these structural phenotypes.

These analyses included 3,488 participants for CVD-PRS and 812 participants for CMR phenotypes. Compared with the full LFI cohort, the PRS subset showed no material differences from the full cohort (all absolute standardized mean differences <0.06), and the CMR subset showed a younger, healthier profile, including lower age and current smoking and higher estimated glomerular filtration rate (standardized mean differences -0.28, -0.15, and 0.23, respectively), with a lower MACE rate (13.7% vs. 22.8%) (**Supplementary Table 7**).

### Incremental predictive value and discriminative performance

**Figure 5:**
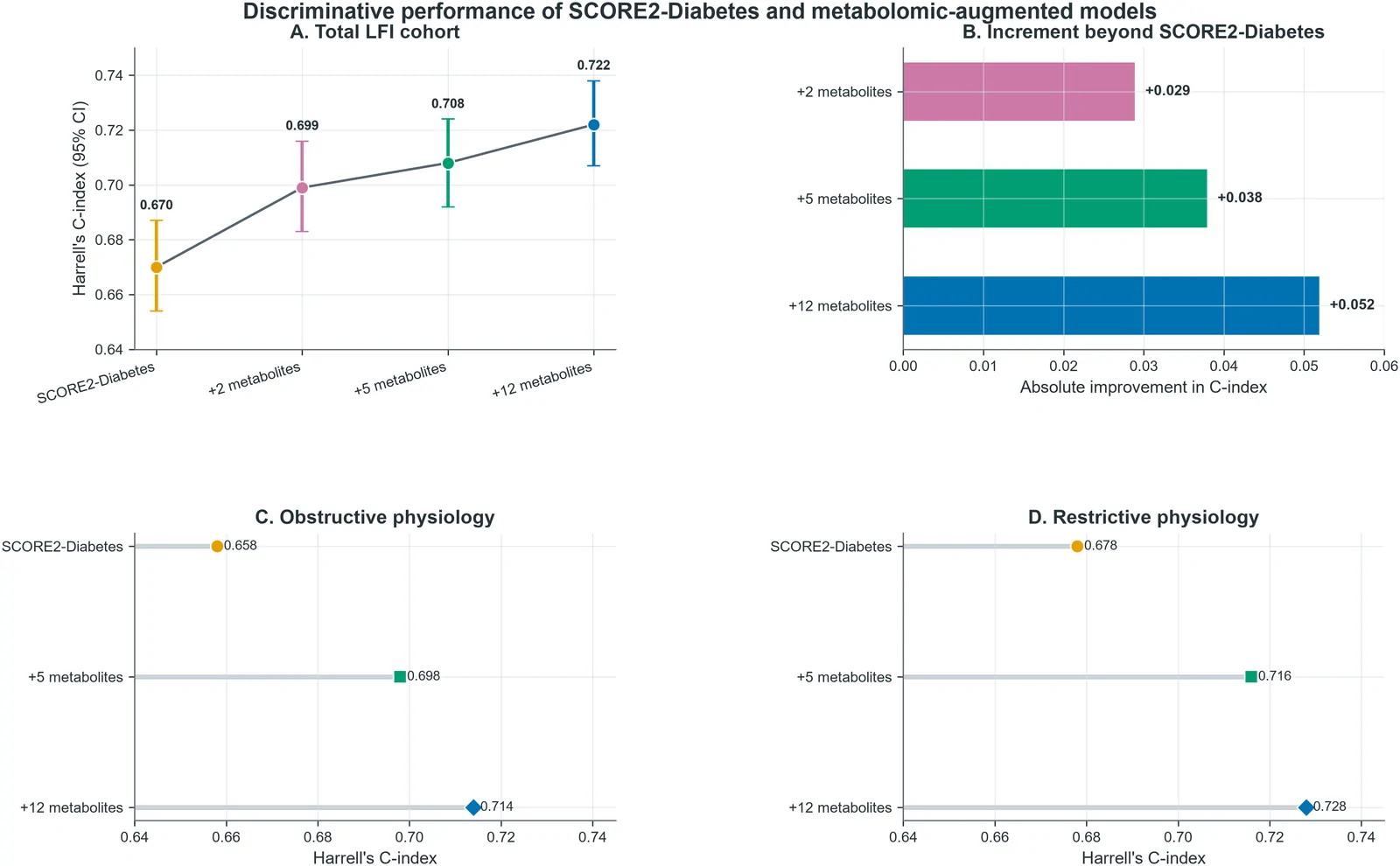
Incremental predictive value and discriminative performance of the metabolomic models. **(A)** Harrell’s C-index and 95% confidence intervals for SCORE2-Diabetes and the two-, five-, and 12-metabolite models in the total LFI cohort. **(B)** Absolute C-index improvement beyond SCORE2-Diabetes in the total LFI cohort. **(C)** C-index values in participants with obstructive physiology. **(D)** C-index values in participants with restrictive physiology. Panels **(C, D)** display point estimates because subgroup confidence intervals were not used in the model-complexity comparison. CI, confidence interval; C-index, concordance index; LFI, lung function impairment; MACE, major adverse cardiovascular events.

We benchmarked the performance of the standard SCORE2-Diabetes model across the study populations to assess its baseline applicability. In the reference group comprising T2D participants with preserved lung function (N = 12,130), the clinical model had a C-index of 0.707 (95% CI: 0.696–0.718). Its discrimination was lower in the study cohort with LFI (N = 3,706), yielding a significantly lower C-index of 0.670 (95% CI: 0.654–0.687) (**Figure 5**).

To examine the trade-off between model complexity and discrimination, we defined a five-metabolite model using the empirical elbow in the ranked importance curve (importance score approximately 0.020) and a two-metabolite sensitivity model containing GlycA and isoleucine, the two dominant predictors (**Figure 3B**). The two-metabolite model achieved a C-index of 0.699 (95% CI, 0.683-0.716), compared with 0.708 (95% CI, 0.692-0.724) for the five-metabolite model and 0.722 (95% CI, 0.707-0.738) for the 12-metabolite model. The corresponding changes versus SCORE2-Diabetes were 0.029, 0.038, and 0.052, respectively. The complete nested-resampling analysis yielded estimates consistent with the prespecified model comparison. In obstructive physiology (**Figure 5C**), the 12-metabolite model increased the C-index from 0.658 to 0.714; in restrictive physiology (**Figure 5D**), it increased from 0.678 to 0.728.

An exploratory subgroup analysis by baseline chronic liver disease status yielded similar findings. Among 286 participants with chronic liver disease (79 MACE), the C-index increased from 0.655 (95% CI, 0.604-0.706) for SCORE2-Diabetes to 0.711 (95% CI, 0.664-0.758) with the 12-metabolite model; corresponding estimates among 3,420 participants without chronic liver disease (766 MACE) were 0.672 (95% CI, 0.655-0.689) and 0.723 (95% CI, 0.708-0.738), with no evidence of heterogeneity (P for interaction=0.71).

### Risk reclassification, calibration, and clinical utility

**Figure 6:**
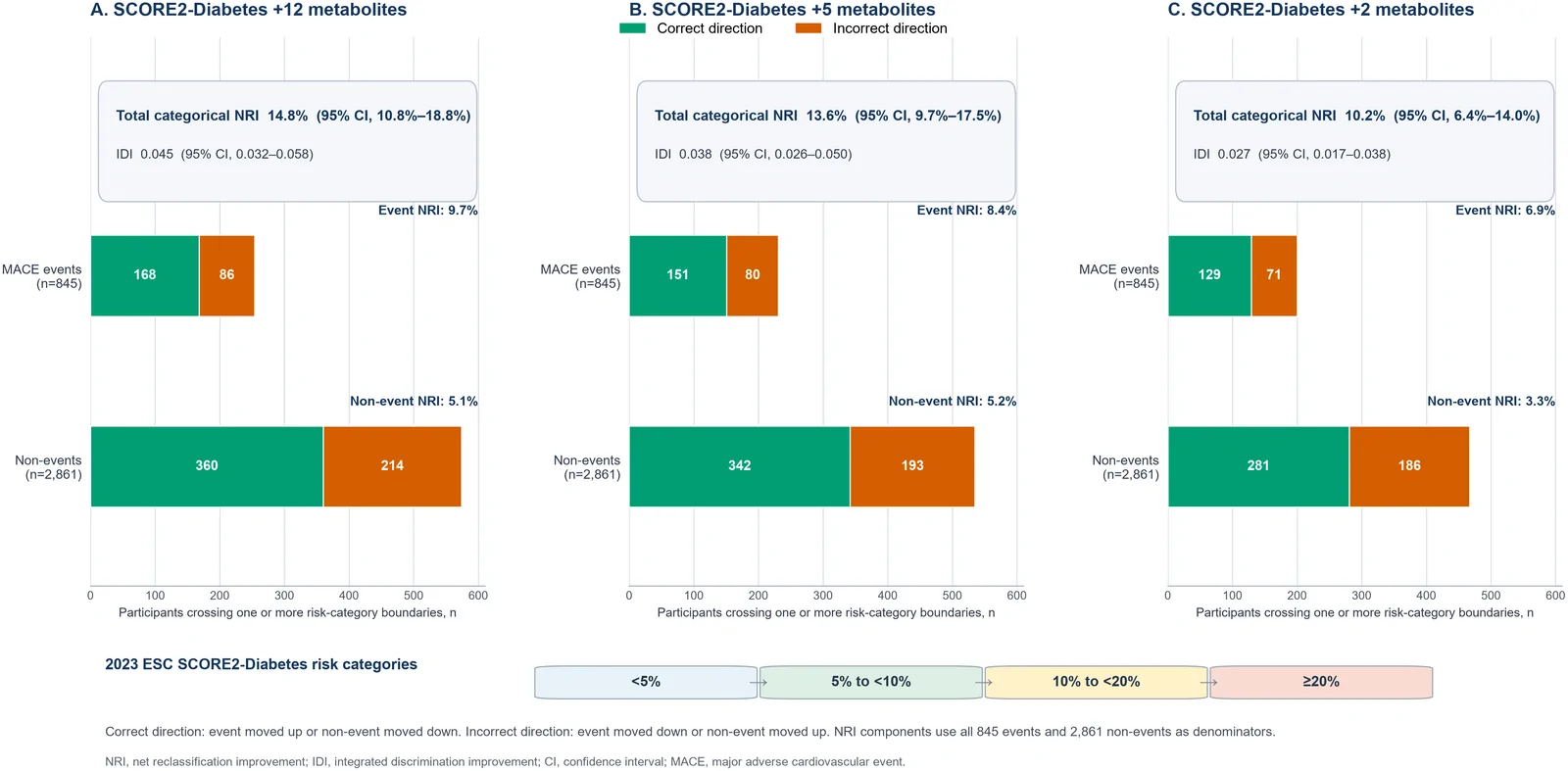
**(A)** SCORE2-Diabetes + 12 metabolites; **(B)** SCORE2-Diabetes + 5 metabolites; **(C)** SCORE2-Diabetes + 2 metabolites. Evaluation of risk reclassification improvement by metabolomic signatures. The reclassification summary shows movement between the 2023 European Society of Cardiology SCORE2-Diabetes categories (<5%, 5% to <10%, 10% to <20%, and ≥20%). For the 12-metabolite model, event NRI, non-event NRI, total NRI, and IDI were 9.7%, 5.1%, 14.8% (95% CI, 10.8%-18.8%), and 0.045 (95% CI, 0.032-0.058), respectively. The corresponding values were 8.4%, 5.2%, 13.6% (95% CI, 9.7%-17.5%), and 0.038 (95% CI, 0.026-0.050) for five metabolites, and 6.9%, 3.3%, 10.2% (95% CI, 6.4%-14.0%), and 0.027 (95% CI, 0.017-0.038) for two metabolites. Correct reclassification denotes an event moving to a higher category or a non-event moving to a lower category; the converse denotes incorrect reclassification. Thresholds support shared decision-making about treatment intensity and do not prescribe a single clinical action. CI, confidence interval.

Using the 2023 European Society of Cardiology SCORE2-Diabetes categories (<5%, 5% to <10%, 10% to <20%, and ≥20%), which support shared decision-making about prevention intensity rather than mandate a single intervention (26), the categorical NRI was 14.8% (95% CI, 10.8%-18.8%) for the 12-metabolite model, 13.6% (95% CI, 9.7%-17.5%) for the five-metabolite model, and 10.2% (95% CI, 6.4%-14.0%) for the two-metabolite model (**Figure 6**, **Supplementary Table 10**). Corresponding IDI estimates were 0.045 (95% CI, 0.032-0.058), 0.038 (95% CI, 0.026-0.050), and 0.027 (95% CI, 0.017-0.038), respectively.

**Figure 7:**
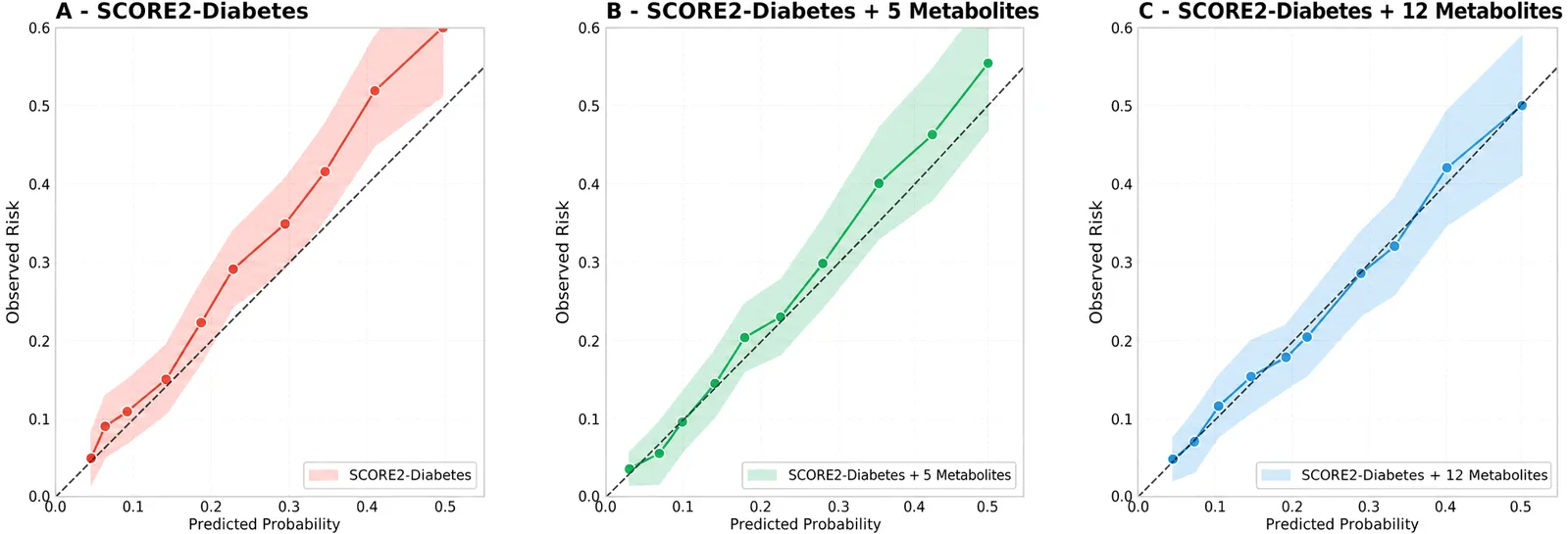
Calibration curves comparing the concordance between predicted probabilities and observed event rates. **(A)** SCORE2-Diabetes. **(B)** SCORE2-Diabetes plus five metabolites. **(C)** SCORE2-Diabetes plus 12 metabolites. Points show observed 10-year risk within deciles of predicted risk; shaded areas indicate 95% confidence intervals. CI, confidence interval.

**Figure 8:**
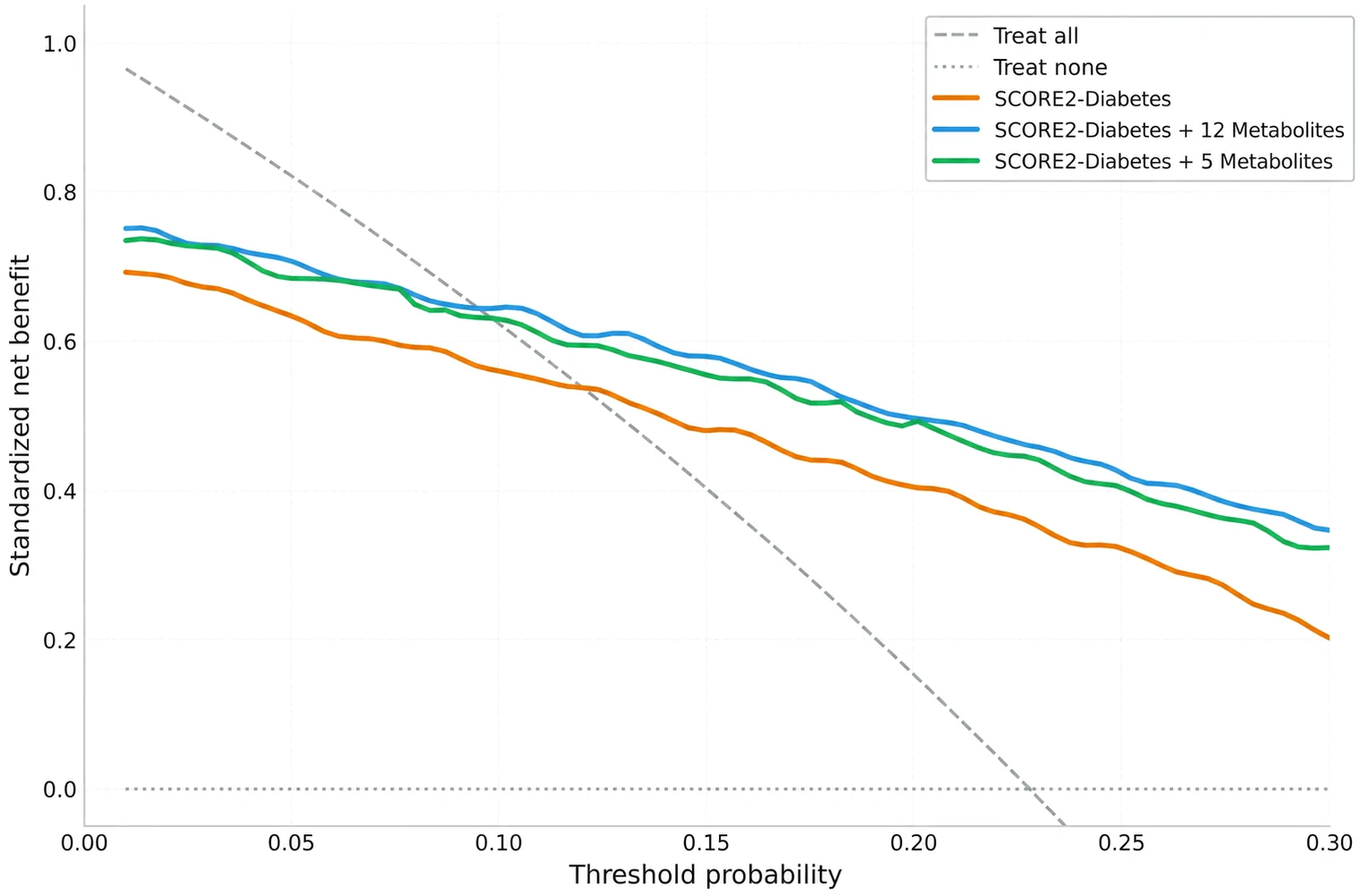
Decision curve analysis of metabolomic-augmented risk models. The curves estimate the standardized net benefit (y-axis) across a range of decision threshold probabilities (x-axis) for predicting 10-year MACE. The performance of the base SCORE2-Diabetes model (orange line) is compared with the Parsimonious Metabo-Model (adding top 5 metabolites, green line) and the Full Metabo-Model (adding 12 metabolites, blue line). The grey dashed line represents the strategy of assuming all patients are at high risk (“Treat all”), while the horizontal dotted line represents assuming no patients are at risk (“Treat none”). DCA, decision curve analysis; MACE, major adverse cardiovascular events.

At 10 years, SCORE2-Diabetes showed evidence of underprediction at higher predicted risks, whereas the metabolomic models showed closer agreement between predicted and observed risk in the internally validated calibration plots (**Figure 7**). Calibration-in-the-large, calibration slopes, and Brier scores were 0.041, 0.84, and 0.158 for SCORE2-Diabetes; 0.019, 0.93, and 0.151 for the two-metabolite model; 0.012, 0.96, and 0.148 for the five-metabolite model; and 0.006, 0.99, and 0.145 for the 12-metabolite model, respectively (**Supplementary Table 9**). In the competing-risk sensitivity analysis, 498 non-cardiovascular deaths were observed and discrimination increased from 0.668 to 0.719 after adding the 12-metabolite signature (**Supplementary Table 11**). The proportional-hazards assumption was satisfied (global P = 0.318; all covariate-specific P>0.05; **Supplementary Table 12**). DCA suggested higher net benefit for the metabolomic models than for SCORE2-Diabetes over threshold probabilities of 1%-30% (**Figure 8**). Because these estimates were obtained in the development cohort, they should be interpreted as internal evidence rather than proof of clinical effectiveness.

## Discussion

In this prospective UK Biobank study of individuals with T2D and LFI, a 12-metabolite signature improved discrimination beyond SCORE2-Diabetes from 0.670 to 0.722 (absolute increase, 0.052). Although statistically significant, this increment is moderate. The reclassification, calibration, and DCA findings provide complementary internal evidence, but they do not by themselves establish that use of the signature would change treatment decisions or improve patient outcomes.

Our findings align with and extend a growing body of literature demonstrating the utility of metabolomics for enhancing CVD risk prediction (29–31). Several recent studies have shown that adding metabolic biomarkers to established risk scores can improve their performance (32–34). For instance, Xie et al. recently demonstrated that adding a 7-metabolite panel to the SCORE2-Diabetes model increased the C-index from 0.67 to 0.71 in a general T2D population from the UK Biobank (32). Similarly, another study by the same group found that metabolomics improved the SCORE2 model in a general population without diabetes, with a C-index increase of approximately 0.04 (33). Other studies have also reported significant, albeit varied, improvements in risk prediction by incorporating metabolomic data (29, 35–37). However, these studies have largely focused on general T2D or non-diabetic populations. The cardiovascular risk landscape in patients with T2D and co-existing LFI is known to be particularly complex and severe (38, 39). A recent landmark study by Chen et al. involving over 16,000 T2D participants from the UK Biobank, found that both obstructive (HR 1.19) and restrictive (HR 1.47) lung physiology were associated with a significantly elevated risk of incident CVD, and that adding spirometry data to the SCORE2-Diabetes model improved its predictive performance (6). To our knowledge, our study is the first to specifically leverage metabolomics to enhance risk stratification within this high-risk T2D-LFI subgroup. By focusing on this vulnerable population, we address a critical predictive gap and observed an absolute C-index increase of 0.052, which is of a similar order to improvements reported in broader populations; its clinical importance remains to be established. Furthermore, our multi-dimensional analysis, linking the metabolic signature not only to incident MACE but also to genetic predisposition (CVD-PRS) and subclinical cardiac remodeling (LVM, RVEDV), provides a more holistic view of the underlying pathophysiology than previous studies.

The selected metabolites offer plausible biological mechanisms linking LFI and CVD risk. GlycA, a composite NMR signal of glycosylated acute-phase proteins, emerged as the top predictor. This finding is consistent with the ‘common soil’ hypothesis, which posits that chronic inflammation serves as the shared pathophysiological antecedent linking metabolic disorders to cardiovascular disease (40, 41). This is consistent with its established role as a robust marker of systemic, chronic inflammation, which is a cornerstone of both atherosclerosis and the pathophysiology of lung disease (42, 43). Elevated GlycA likely reflects the heightened inflammatory state in our cohort, providing a plausible molecular link between pulmonary inflammation and cardiovascular pathology (44, 45). The strong association of branched-chain amino acids (BCAAs) like isoleucine, leucine, and valine with MACE risk points towards the role of insulin resistance and metabolic dysfunction (46, 47). Dysregulation of BCAA metabolism is known to promote mitochondrial dysfunction, oxidative stress, and macrophage activation, all of which are key processes in the development of atherosclerosis (48, 49). Lactate, a central product of glycolysis, may reflect a shift towards anaerobic metabolism, which is often observed in states of tissue hypoxia and heart failure (50, 51). Conversely, albumin, which was a protective factor in our model, is known for its antioxidant, anti-inflammatory, and anti-thrombotic properties, and low serum albumin is a well-documented predictor of cardiovascular mortality (52, 53).

From a clinical perspective, the five-metabolite panel is analytically simpler than the 12-metabolite panel, but assay feasibility, standardization, cost-effectiveness, and clinical impact were not evaluated in this study. If externally validated, such a panel could be studied as an adjunct for identifying patients who may warrant a clinician-patient discussion about treatment intensity, including established therapies such as sodium-glucose cotransporter 2 (SGLT2) inhibitors or glucagon-like peptide-1 (GLP-1) receptor agonists (54, 55). The internally observed calibration and DCA improvements are encouraging but threshold-dependent. A C-index increase of 0.052 and statistically significant reclassification do not necessarily translate into clinically important benefit; independent validation, decision-impact studies, and formal economic evaluation are required before implementation.

This study has several notable strengths, including its large-scale, well-characterized prospective design based on the UK Biobank, the utilization of a standardized, high-throughput NMR platform for metabolomic profiling, and a rigorous statistical framework employing multiple machine learning algorithms and nested internal validation. The multi-dimensional validation against genetic risk scores and cardiac imaging data further enhances the biological plausibility and internal consistency of our findings. However, several limitations should be considered. First, while the prospective nature of our cohort study establishes a temporal sequence between metabolic signatures and incident MACE, the inherent observational design means that the identified associations should be interpreted with caution regarding definitive causality. Second, the UK Biobank participants are predominantly of European ancestry and tend to be healthier than the general population (the “healthy volunteer” effect), which may limit the generalizability of our predictive models to more diverse ethnic groups or different clinical settings (56). Third, although we adjusted for a comprehensive set of established cardiovascular risk factors and potential confounders, the possibility of residual confounding from unmeasured variables cannot be entirely excluded.

Most importantly, the model has not undergone independent external validation. The UK Biobank’s non-random participation, predominantly European ancestry, and healthy-volunteer selection may affect both baseline risk and predictor distributions, limiting transportability. Selection into the CVD-PRS and CMR subsets may introduce additional bias, as summarized in **Supplementary Table 7**. Finally, the apparent clinical utility is based on internal reclassification and DCA; prospective studies are needed to determine whether model-guided care changes management or outcomes.

## Conclusion

In conclusion, the 12-metabolite signature produced a statistically significant but moderate improvement in cardiovascular risk prediction among UK Biobank participants with T2D and LFI. The biological associations support plausibility, while independent external validation and prospective clinical-impact evaluation remain necessary before the model can be considered for practice.

## Data Availability

The data analyzed in this study are available from UK Biobank, subject to its access procedures and approval. The datasets are not publicly available from the authors because they were used under license. Applications may be submitted through the UK Biobank Access Management System.
