# Supplementary material for: Metabolomic profiling refines cardiovascular risk stratification beyond SCORE2-diabetes in patients with concurrent type 2 diabetes and pulmonary dysfunction
Source: Front Endocrinol (Lausanne). 2026 Aug 26;17:1933396. doi: 10.3389/fendo.2026.1933396 (PMC13553505; doi:10.3389/fendo.2026.1933396)
Supplement: Supplementary file 1 [file DataSheet1.pdf]

Supplemental Materials to

Metabolomic Profiling Refines Cardiovascular Risk Stratification Beyond SCORE2-Diabetes in Patients with Concurrent Type 2 Diabetes and Pulmonary Dysfunction

Table of Contents

Supplementary Methods *2*

Supplementary Table S1. Definitions and UK Biobank field IDs for defining type 2 diabetes *5*

Supplementary Table S2. Definitions of lung function and lung function impairment *6*

Supplementary Table S3. Definition of endpoint major cardiovascular event (MACE) *7*

Supplementary Table S4. Missing values for covariates in the study cohort (N=3,706) *8*

Supplementary Table S5. List of metabolites selected by three machine learning methods *9*

Supplementary Table S6. β-coefficients of the variables of the SCORE2-Diabetes model extended by selected metabolites *11*

Supplementary Table S7. Comparison of the CVD-PRS and CMR subsets with the full LFI cohort *12*

Supplementary Table S8. Bootstrap selection frequency of the 12 consensus metabolites *13*

Supplementary Table S9. Nested-resampling discrimination and calibration performance *14*

Supplementary Table S10. Categorical NRI and IDI for the metabolomic models *15*

Supplementary Table S11. Competing-risk sensitivity analysis *16*

Supplementary Table S12. Proportional-hazards tests *17*

Supplementary Table S13. Nested-resampling and hyperparameter specifications *18*

Supplementary Figure S1. Flowchart of study participant selection *19*

Supplementary Methods

1. Assessment of Lung Function and Quality Control

Spirometric assessments were conducted at baseline assessment centers using a Vitalograph Pneumotrac 6800 spirometer (Vitalograph Ltd., Maids Moreton, UK) in strict adherence to the standardized protocols outlined by the American Thoracic Society and European Respiratory Society (ATS/ERS) [1]. Prior to testing, participants were screened for contraindications, including recent chest infections, ophthalmic or thoracic surgeries, and current pregnancy, to ensure safety and data validity. Participants were instructed to perform up to three forced expiratory maneuvers to achieve maximal effort. To minimize measurement error and ensure high data quality, the "best measure" for analysis was defined as the highest Forced Expiratory Volume in 1 second (FEV1) and Forced Vital Capacity (FVC) derived from technically acceptable curves that were free from artifacts such as coughing, glottis closure, or early termination. To account for physiological variations across different demographic groups, we utilized the Global Lung Function Initiative (GLI) 2012 multi-ethnic reference equations [2]. Unlike traditional fixed-ratio criteria, the GLI 2012 equations employ sophisticated spline-based modeling to derive age-, sex-, height-, and ethnicity-specific Lower Limits of Normal (LLN), thereby providing a more accurate physiological threshold for defining impairment. Consequently, lung function phenotypes were strictly categorized based on these LLN thresholds: preserved lung function was defined as both FEV1/FVC and FVC ≥ LLN; obstructive physiology was defined as an FEV1/FVC ratio < LLN; and restrictive physiology was characterized by an FEV1/FVC ratio ≥ LLN with a concomitant FVC < LLN.

References:

[1] Miller MR, et al. Standardisation of spirometry. *Eur Respir J*. 2005;26(2):319-338.
[2] Quanjer PH, et al. Multi-ethnic reference values for spirometry for the 3-95-yr age range: the global lung function 2012 equations. *Eur Respir J*. 2012;40(6):1324-1343.

2. Profiling of Plasma Metabolomics

Circulating metabolic biomarkers were quantified in baseline EDTA-plasma samples using a high-throughput proton nuclear magnetic resonance (^{1}H-NMR) spectroscopy platform provided by Nightingale Health Ltd. (Helsinki, Finland). This targeted metabolomics platform facilitates the simultaneous quantification of 249 metabolic measures, comprising 168 absolute concentrations and 81 derived ratios, from a single plasma aliquot [3]. The comprehensive panel covers a diverse array of metabolic pathways, including detailed lipoprotein subclass profiling (particle size, density, and lipid composition across 14 subclasses ranging from VLDL to HDL), fatty acid composition (total fatty acids, saturated,

monounsaturated, and polyunsaturated fatty acids including omega-3 and omega-6), and abundant low-molecular-weight metabolites such as amino acids (e.g., alanine, glutamine, branched-chain amino acids), ketone bodies (acetate, acetoacetate, 3-hydroxybutyrate), and glycolysis-related metabolites (glucose, lactate, citrate). Additionally, the platform quantifies key inflammatory and fluid balance markers, specifically glycoprotein acetyls (GlycA), albumin, and creatinine. The analytical process involves minimal sample preparation with no extraction steps, ensuring high reproducibility and minimizing technical variability [4]. Samples were mixed with a phosphate buffer prior to spectral acquisition, and automated quality control procedures were applied to monitor line width, baseline irregularities, and sample degradation. For the current analysis, metabolites with greater than 20% missingness or those falling below the limit of detection were excluded to maintain the robustness of the dataset.

References:

[3] Soininen, P., Kangas, A. J., Würtz, P., Suna, T., & Ala-Korpela, M. (2015). Quantitative serum nuclear magnetic resonance metabolomics in cardiovascular epidemiology and genetics. *Circulation. Cardiovascular genetics*, *8*(1), 192–206.

[4] Würtz, P., Havulinna, A. S., Soininen, P., Tynkkynen, T., Prieto-Merino, D., Tillin, T., Ghorbani, A., Artati, A., Wang, Q., Tiainen, M., Kangas, A. J., Kettunen, J., Kaikkonen, J., Mikkilä, V., Jula, A., Kähönen, M., Lehtimäki, T., Lawlor, D. A., Gaunt, T. R., Hughes, A. D., … Salomaa, V. (2015). Metabolite profiling and cardiovascular event risk: a prospective study of 3 population-based cohorts. *Circulation*, *131*(9), 774–785.

3. Calculation of SCORE2-Diabetes Risk

The SCORE2-Diabetes risk score was employed to establish a clinical baseline for cardiovascular risk prediction. This algorithm is a specifically calibrated extension of the SCORE2 model, developed by the SCORE2-Diabetes Working Group to estimate the 10-year risk of fatal and non-fatal cardiovascular disease (CVD) (defined as myocardial infarction or stroke) in individuals with type 2 diabetes [5]. The model integrates conventional CVD risk factors—including age at recruitment, sex, smoking status, systolic blood pressure (mean of two automated measurements), total cholesterol, and high-density lipoprotein cholesterol (HDL-C)—with diabetes-specific parameters, namely glycated hemoglobin (HbA1c), estimated glomerular filtration rate (eGFR) calculated via the CKD-EPI 2009 creatinine equation, and age at diabetes diagnosis. To ensure accurate risk estimation appropriate for the study population, we utilized the recalibrated risk equations corresponding to the "Moderate Risk" region, which is the designated classification for the United Kingdom within the European Society of Cardiology (ESC) guidelines. The final score represents the

absolute percentage probability of a participant developing a CVD event within the subsequent 10 years, serving as a robust comparator for assessing the incremental predictive value of novel metabolic markers.

References:

[5] SCORE2-Diabetes Working Group and the ESC Cardiovascular Risk Collaboration. SCORE2-Diabetes: 10-year cardiovascular risk estimation in type 2 diabetes in Europe. *Eur Heart J*. 2023;44(28):2544-2556.

4. Nested Resampling, Hyperparameter Tuning, and Consensus Feature Selection

The entire analysis pipeline was repeated de novo within each of 1,000 bootstrap resamples. Within every resample, missing-data imputation and metabolite standardization were estimated from the bootstrap sample, and hyperparameters were tuned by inner 10-fold cross-validation. LASSO used the one-standard-error rule. Random-forest and XGBoost hyperparameters were selected from the grids reported in Supplementary Table S13. Feature selection by LASSO, random forest, and XGBoost was repeated within the resample, after which the prespecified intersection rule was applied and the Cox model was fitted. Predictive performance was evaluated in out-of-bag participants and aggregated across resamples. The intersection was chosen as a conservative consensus rule that reduces method-specific selection, while recognizing that signals selected by only one or two algorithms may be missed.

5. Competing-Risk and Calibration Analyses

Primary Cox models censored non-cardiovascular deaths at the date of death. Sensitivity analyses treated non-cardiovascular death as a competing event using Fine–Gray regression and the Aalen–Johansen estimator. Ten-year calibration was evaluated in deciles of predicted risk. Calibration-in-the-large, the calibration slope, and the Brier score were estimated with bootstrap correction. Decision-curve analysis examined threshold probabilities from 1% to 30%.

6. Model Diagnostics and Subset Comparisons

The proportional-hazards assumption was assessed using scaled Schoenfeld residuals for each covariate and a global test. Participants included in the cardiovascular disease polygenic risk score and cardiac magnetic resonance analyses were compared with the full lung-function-impairment cohort using standardized mean differences.

Supplementary Table S1. Definitions and UK Biobank field IDs for defining type 2 diabetes

**Description**	**Field ID**	**Definitions**
Type 2 diabetes at baseline		Participants were defined as having T2D if they met one or more of the following criteria:
Age diabetes diagnosed	2976	Age at diagnosis was less than or equal to age at assessment.
Diabetes diagnosed by doctor	2443	Answered "Yes" to the question: "Has a doctor ever told you that you have diabetes?"
Non-cancer illness code, self-reported	20002	Self-reported medical history of type 2 diabetes (code "1223").
Medication for cholesterol, blood pressure, diabetes	6153, 6177	Taking anti-diabetic medications (including insulin).
Treatment/medication code	20003	Use of insulin or oral hypoglycemic medication.
Date T2D first reported (ICD-10)	130708	Date of first report for ICD-10 code E11 (Non-insulin-dependent diabetes mellitus) was before or at the date of assessment.
Glycated hemoglobin (HbA_{1c})	30750	HbA_{1c} level ≥ 48 mmol/mol (6.5%).
Hospital episode records	41270, 41280	Hospitalization record with ICD-10 code E11, with a date before or at the date of assessment.

Supplementary Table S2. Definitions of lung function and lung function impairment

Category	Field ID	Definition
**Spirometry Measures**		
FEV1, Best measure	20151	Best measure of FEV1 from technically acceptable maneuvers.
FVC, Best measure	20150	Best measure of FVC from technically acceptable maneuvers.
**Derived Categories**		
Preserved Lung Function	-	FEV1/FVC ratio ≥ LLN and FVC ≥ LLN.
Obstructive Physiology	-	FEV1/FVC ratio < LLN. This indicates airflow limitation.
Restrictive Physiology	-	FEV1/FVC ratio ≥ LLN and FVC < LLN. This suggests a reduction in lung volume.

Abbreviations: FEV1, forced expiratory volume in 1 second; FVC, forced vital capacity; LLN, lower limit of normal; GLI, Global Lung Function Initiative.

Supplementary Table S3. Definition of endpoint major cardiovascular event (MACE)

**Fatal MACE – cause-specific mortality due to any of the following:**	
Endpoints included	ICD10-codes
Hypertensive disease	I10-16
Ischemic heart disease	I20-25
Arrhythmias, heart failure	I46-52
Cerebrovascular disease	I60-69
Atherosclerosis/aortic aneurysm	I70-73
Sudden death and death within 24 hours of symptom onset	R96.0-96.1
Endpoints excluded from the above endpoint:	ICD10-codes
Myocarditis, unspecified	I51.4
Subarachnoid haemorrhage	I60
Subdural hemorrhage	I62
Cerebral aneurysm	I67.1
Cerebral arteritis	I68.2
Moyamoya	I67.5
**Non-fatal MACE**	ICD10-codes
Non-fatal myocardial infarction	I21-I23
Non-fatal stroke	I61, I63-I66, I69

The primary endpoint was a composite of the first occurrence of a major adverse cardiovascular event (MACE), including non-fatal myocardial infarction (MI), non-fatal stroke, or cardiovascular death. Event data were ascertained from hospital inpatient records and death registries.

Supplementary Table S4. Missing values for covariates in the study cohort (N=3,706)

Variable	Missing Count (N)	Missing Percentage (%)
**Demographics & Lifestyle**		
BMI, kg/m²	34	0.9%
Current Smoker, n (%)	11	0.3%
Clinical Measures		
Systolic BP, mmHg	45	1.2%
Diastolic BP, mmHg	45	1.2%
HbA_{1c}, mmol/mol	31	0.8%
eGFR, mL/min/1.73m²	62	1.7%
Total Cholesterol, mmol/L	38	1.0%
HDL-C, mmol/L	123	3.3%
Triglycerides (clinical), mmol/L	49	1.3%
Age at diabetes diagnosis	45	1.2%
**Metabolites (per 1-SD)**		
GlycA	0	0.0%
Isoleucine	0	0.0%
Lactate	0	0.0%
Albumin	0	0.0%
HDL Size	0	0.0%
Glucose	0	0.0%
Triglycerides (NMR)	0	0.0%
Glutamine	0	0.0%
Histidine	0	0.0%
Leucine	0	0.0%
Valine	0	0.0%
Citrate	0	0.0%

Abbreviations: HbA1c, glycated hemoglobin; HDL, high-density lipoprotein; SBP, systolic blood pressure; SD, standard deviation.

Supplementary Table S5. List of metabolites selected by three machine learning methods

LASSO (n=34)	Random Forest (n=42)	XGBoost (n=37)
GlycA	GlycA	GlycA
Isoleucine	Isoleucine	Isoleucine
Lactate	Lactate	Lactate
Albumin	Albumin	Albumin
HDL Size	HDL Size	HDL Size
Glucose	Glucose	Glucose
Triglycerides	Triglycerides	Triglycerides
Glutamine	Glutamine	Glutamine
Histidine	Histidine	Histidine
Leucine	Leucine	Leucine
Valine	Valine	Valine
Citrate	Citrate	Citrate
Pyruvate	Phenylalanine	Tyrosine
Alanine	Total fatty acids	Linoleic acid
Omega-6 fatty acids	VLDL particle size	Acetoacetate
3-hydroxybutyrate	Glycine	Total cholines
Phosphatidylcholines	LDL particle size	Total BCAA
Creatinine	Acetate	Phospholipids
Apolipoprotein B	Free cholesterol	Esterified cholesterol
Sphingomyelins	Docosahexaenoic acid	Omega-3 fatty acids
Monounsaturated fatty acids	Polyunsaturated fatty acids	HDL cholesterol
LDL cholesterol	IDL particle concentration	Remnant cholesterol
Total BCAA	Tyrosine	Alanine
Phenylalanine	Pyruvate	VLDL particle size
Glycine	Acetoacetate	3-hydroxybutyrate
Acetate	Total cholines	LDL particle size

Phospholipids	Phosphatidylcholines	Creatinine
Free cholesterol	Sphingomyelins	Apolipoprotein B
Esterified cholesterol	Monounsaturated fatty acids	Free cholesterol
Docosahexaenoic acid	HDL cholesterol	Esterified cholesterol
Omega-3 fatty acids	LDL cholesterol	Sphingomyelins
Polyunsaturated fatty acids	Remnant cholesterol	Docosahexaenoic acid
IDL particle concentration	Total BCAA	Omega-3 fatty acids
Remnant cholesterol	Glycine	Polyunsaturated fatty acids
	Phenylalanine	IDL particle concentration
	Alanine	HDL cholesterol
	Linoleic acid	LDL cholesterol
	Omega-6 fatty acids	
	Creatinine	
	Apolipoprotein B	

Cells shaded in gray indicate the 12-metabolite signature identified by the intersection of all three methods.

Supplementary Table S6. β-coefficients of the variables of the SCORE2-Diabetes model extended by selected metabolites

**Risk factor (units)**	**SCORE2-Diabetes + 12 Metabolites**	**SCORE2-Diabetes + 5 Metabolites**
**SCORE2-Diabetes variables**		
Age (per 5 years)	0.485	0.501
Current smoking	0.155	0.162
SBP (per 20 mmHg)	0.110	0.115
Total cholesterol (per 1 mmol/L)	0.082	0.091
HDL cholesterol (per 0.5 mmol/L)	-0.185	-0.199
Age at diabetes diagnosis (per 5 years)	-0.077	-0.081
HbA_{1c} (per 10 mmol/mol)	0.121	0.128
Ln eGFR (per 1 SD)	-0.153	-0.160
**Interaction Terms**		
Smoking interaction with age	-0.041	-0.045
SBP interaction with age	0.095	0.102
Total cholesterol interaction with age	-0.058	-0.063
HDL interaction with age	0.021	0.025
**Additional metabolites (per 1 SD)**		
GlycA	0.299	0.315
Isoleucine	0.251	0.268
Lactate	0.188	0.201
Albumin	-0.215	-0.224
HDL Size	-0.150	-0.165
Glucose	0.135	-
Triglycerides	0.129	-
Glutamine	-0.111	-
Histidine	-0.105	-
Leucine	0.101	-
Valine	0.098	-
Citrate	-0.095	-

Supplementary Table S7. Comparison of the CVD-PRS and CMR subsets with the full LFI cohort

**Characteristic**	**Full LFI cohort (N=3,706)**	**CVD-PRS subset (N=3,488)**	**CMR subset (N=812)**
Age, years	66.3 (7.9)	66.2 (7.8)	64.1 (7.1)
Male	65.0%	64.8%	62.1%
Current smoking	10.1%	10.0%	5.9%
Systolic blood pressure, mmHg	136.6 (16.7)	136.5 (16.6)	133.8 (15.4)
HbA1c, mmol/mol	54.6 (10.6)	54.5 (10.5)	52.9 (9.8)
eGFR, mL/min/1.73 m²	80.3 (20.2)	80.5 (20.0)	85.0 (18.4)
Obstructive physiology	33.1%	33.0%	30.5%
MACE	845 (22.8%)	797 (22.9%)	111 (13.7%)
Maximum |SMD| vs. full cohort	—	<0.06	0.28

Values are mean (standard deviation), n (%), or percentage unless otherwise indicated. CMR, cardiac magnetic resonance; CVD-PRS, cardiovascular disease polygenic risk score; LFI, lung function impairment; MACE, major adverse cardiovascular event; SMD, standardized mean difference.

Supplementary Table S8. Bootstrap selection frequency of the 12 consensus metabolites

**Metabolite**	**Selection frequency**
GlycA	98.7%
Isoleucine	96.9%
Lactate	85.4%
Albumin	82.8%
HDL size	80.7%
Glucose	75.9%
Triglycerides	72.6%
Glutamine	68.3%
Histidine	66.1%
Leucine	62.8%
Valine	60.9%
Citrate	58.7%

Selection frequency is the percentage of the 1,000 bootstrap resamples in which the metabolite was retained after resample-specific preprocessing, tuning, and feature selection.

Supplementary Table S9. Nested-resampling discrimination and calibration performance

Model	C-index (95% CI)	Absolute ΔC	Calibration-in-the-large	Calibration slope	Brier score
SCORE2-Diabetes	0.670 (0.654-0.687)	Reference	0.041 (0.018-0.064)	0.84 (0.76-0.92)	0.158 (0.151-0.165)
+ 2 metabolites	0.699 (0.683-0.716)	0.029	0.019 (-0.004-0.042)	0.93 (0.84-1.02)	0.151 (0.144-0.158)
+ 5 metabolites	0.708 (0.692-0.724)	0.038	0.012 (-0.011-0.035)	0.96 (0.87-1.05)	0.148 (0.141-0.155)
+ 12 metabolites	0.722 (0.707-0.738)	0.052	0.006 (-0.017-0.029)	0.99 (0.90-1.08)	0.145 (0.138-0.152)

Calibration measures were assessed at 10 years using deciles of predicted risk and the Aalen–Johansen estimator for observed cumulative incidence. CI, confidence interval.

Supplementary Table S10. Categorical NRI and IDI for the metabolomic models

Model	Event up/down	Event NRI	Non-event down/up	Non-event NRI	Total NRI (95% CI)	IDI (95% CI)
+ 12 metabolites	168 / 86	9.7%	360 / 214	5.1%	14.8% (10.8%-18.8%)	0.045 (0.032-0.058)
+ 5 metabolites	151 / 80	8.4%	342 / 193	5.2%	13.6% (9.7%-17.5%)	0.038 (0.026-0.050)
+ 2 metabolites	129 / 71	6.9%	281 / 186	3.3%	10.2% (6.4%-14.0%)	0.027 (0.017-0.038)

Risk categories were <5%, 5% to <10%, 10% to <20%, and ≥20%. For events, upward movement is correct and downward movement is incorrect; for non-events, downward movement is correct and upward movement is incorrect. IDI, integrated discrimination improvement; NRI, net reclassification improvement.

Supplementary Table S11. Competing-risk sensitivity analysis

**Measure**	**Result**
Non-cardiovascular deaths	498 (13.4%)
10-year MACE cumulative incidence	20.3% (95% CI, 19.0%-21.6%)
Competing-risk C-index: SCORE2-Diabetes	0.668
Competing-risk C-index: +12 metabolites	0.719
GlycA sHR	1.31 (1.22-1.41)
Isoleucine sHR	1.25 (1.16-1.35)
Lactate sHR	1.18 (1.10-1.27)
Albumin sHR	0.83 (0.76-0.91)
HDL size sHR	0.88 (0.82-0.95)

Non-cardiovascular death was treated as a competing event. sHR, subdistribution hazard ratio.

Supplementary Table S12. Proportional-hazards tests

**Test/covariate**	**P value**
Global test	0.318
GlycA	0.482
Isoleucine	0.337
Lactate	0.216
Albumin	0.604
HDL size	0.118
Glucose	0.451
Triglycerides	0.391
Glutamine	0.284
Histidine	0.168
Leucine	0.526
Valine	0.742
Citrate	0.309
Lowest clinical-covariate P value: SBP×age	0.071

P values were obtained from scaled Schoenfeld residual tests. SBP, systolic blood pressure.

Supplementary Table S13. Nested-resampling and hyperparameter specifications

Component	Specification
Bootstrap	1,000 resamples; full pipeline repeated within each resample; out-of-bag evaluation
Inner validation	10-fold cross-validation
LASSO	λ selected by the one-standard-error rule
Random forest	mtry {8, 16, 24, 32, 48}; minimum node size {5, 10, 20}; 1,000 trees
XGBoost	max_depth {2, 3, 4}; eta {0.01, 0.05, 0.10}; min_child_weight {1, 5, 10}; subsample {0.7, 0.9, 1.0}; colsample_bytree {0.6, 0.8, 1.0}; ≤1,000 rounds with early stopping
Random seed	20260802

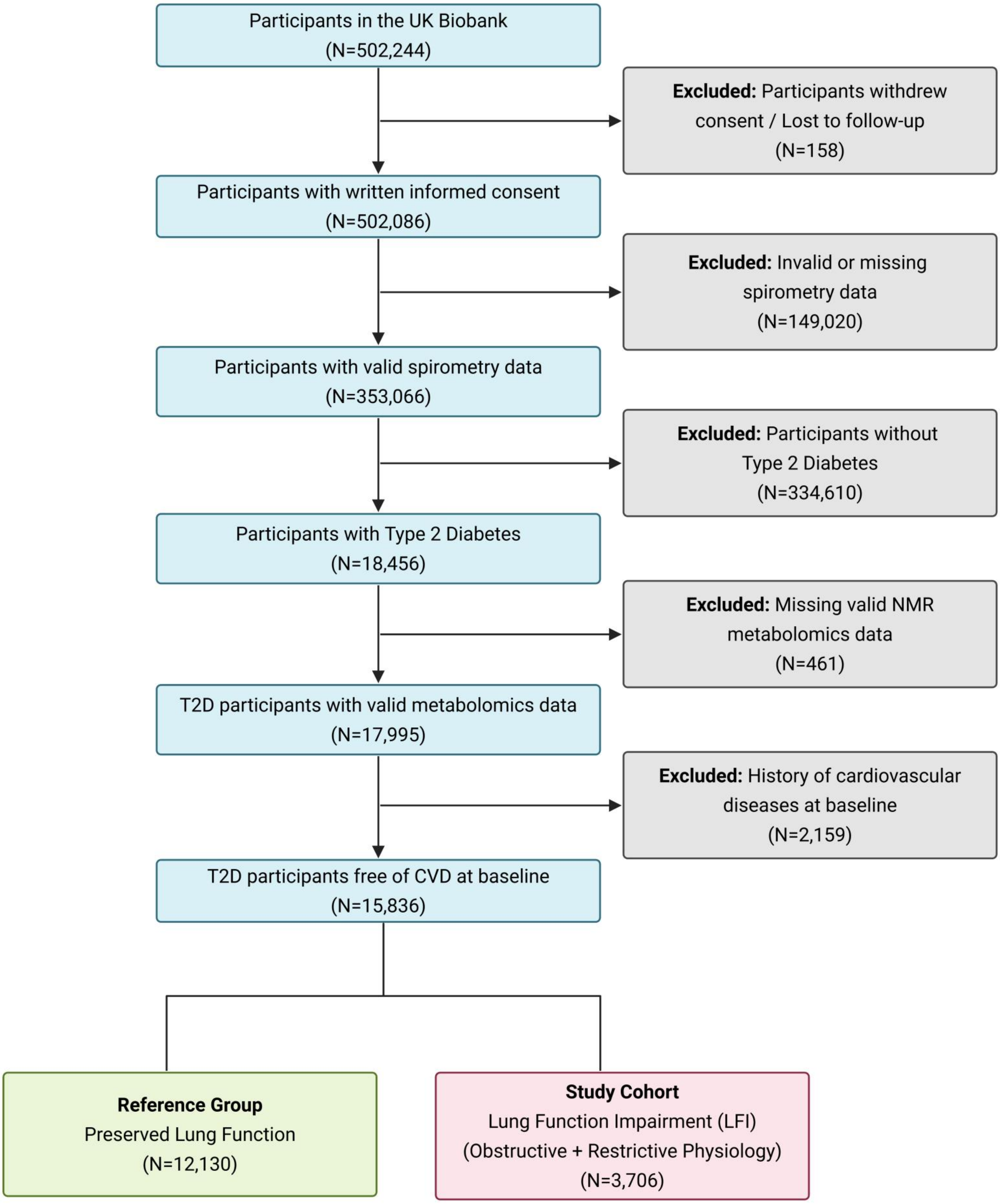

Supplementary Figure S1. Flowchart of study participant selection
